# Impaired Cerebral Vasomotor Reactivity in Alzheimer's Disease

**DOI:** 10.1155/2018/9328293

**Published:** 2018-09-09

**Authors:** Fernando Gongora-Rivera, Adolfo Cordero-Perez, Alejandro Gonzalez-Aquines, Antonio Anaya-Escamilla, Eduardo Villarreal-Garza, Meztli Espinosa-Ortega, Mario C. Salinas-Carmona, Xochilt Ortiz-Jimenez

**Affiliations:** ^1^Departamento de Neurología, “Dr. José Eleuterio González” Hospital Universitario y Facultad de Medicina, Universidad Autónoma de Nuevo León, Monterrey, Mexico; ^2^Unidad de Neuromodulación y Plasticidad Cerebral, Centro de Investigación y Desarrollo en Ciencias de la Salud, Universidad Autónoma de Nuevo León (UANL), Monterrey, NL, Mexico; ^3^Facultad de Psicología, Universidad Autónoma de Nuevo León (UANL), Monterrey, NL, Mexico

## Abstract

**Background:**

Recent studies have shown that cerebral vascularity may be impaired in Alzheimer's disease. Cerebral vasomotor reactivity could be an important biomarker for this pathology.

**Aims:**

The aim of this study was to investigate the alterations in cerebral vascular motor reactivity in Alzheimer's disease subjects and to associate these changes with their cognitive scores.

**Methods:**

We recruited subjects with a diagnosis of Alzheimer's disease and healthy controls. Demographic, clinical, imaging, and cognitive test were obtained. Then all participants performed a cerebral vascular motor reactivity test with 7% CO2 and cerebral blood flow velocities (CBFV) were recorded with transcranial doppler ultrasound before and after the test.

**Results:**

We recruited 45 subjects, 26 (21 female) Alzheimer's disease participants and 19 (15 female) healthy controls. There were no differences in baseline cerebral blood flow velocities between the groups. After the cerebral vasomotor reactivity test, absolute mean difference in mean CBFV (ΔCBFV-m) was 8.70±4.14 versus 4.81±6.96 (p<0.01), respectively. Calculated percentage of change (%CVMR) was lower in the AD group 7.45±18.25 versus 23.29±17.48, and there was a positive but weak correlation with mini-mental scores (*ρ*=0.337, p=0.023).

**Conclusions:**

In this study, Alzheimer's disease subjects showed significant changes in all absolute cerebral blood flow velocities after the cerebral vasomotor reactivity test with CO2, but only diastolic phase responses were statistically significant. There was a positive but weak correlation between cerebral vasomotor reactivity and cognitive scores. Further studies are needed to investigate these effects in larger Latin-American samples.

## 1. Introduction

Alzheimer's disease (AD) is the leading cause of dementia in both industrialized and developing countries, accounting for most of all dementia cases [1, 2]. Furthermore, AD affects an estimate of 46.8 million people worldwide, approximately 5.2% of the world population, and it is calculated that this number will double by the year 2030 [3]. Therefore, it is important to develop cost-effective treatment and prevention strategies. However, the pathophysiological processes behind neurodegeneration in AD remain unclear. Neither an effective treatment or a prevention strategy have been developed.

It is well known that amyloid-*β* protein deposits are the classical pathological finding in subjects affected with AD. These protein deposits are spread across the central nervous system including the brain vessel walls, leading to impaired endothelial function and blood-brain barrier disruption among other pathological phenomena [4]. On the other hand, epidemiological studies have demonstrated that cardiovascular disease (CVD) represents a risk factor for AD development and progression. Common CVD risk factors as hypertension, diabetes mellitus, and dyslipidemia are associated with increased AD frequency [5]. Particularly, participants with peripheral arterial disease (PAD) have a relative risk (RR) of 2.5 to develop AD, relative to those without PAD [6]. Thus, cardiovascular disease may promote and contribute to the development of AD, and in a parallel way, AD could also contribute to vascular damage and cerebral hypoperfusion progression through cerebral amyloid angiopathy and other mechanisms [7].

In normal subjects, the neurovascular unit (NVU), which is a functional unit consisting of neuronal, glial, and vascular cells, is responsible for maintaining an adequate cerebral blood flow (CBF) in response to internal and external stimuli. The NVU (and CBF in consequence) can respond to changes in blood CO2 concentration, which is the termed cerebral vascular motor reactivity or cerebral vasomotor reactivity (CVMR) [4]. Hence CVMR arises as an important biomarker of CBF regulation and NVU function, and in consequence of cerebral vascular health [8]. CVMR attenuation along with vascular structural alterations had been evidenced in several preclinical studies with animal models of AD [9] and human brain specimen studies [10]. Measurement of CBF changes in humans has been done previously using BOLD-MR, SPECT, and PET and indirectly with transcranial doppler ultrasonography (TCD). The latter is a dynamic noninvasive method that measures cerebral blood flow velocity (CBFV) in the brain vessels, most frequently in the middle cerebral artery (MCA).

The aim of this study was to measure and compare CVMR response with TCD in AD participants versus control subjects matched for age and sex and to associate these changes with their cognitive scores in a Latin-American AD sample.

## 2. Materials and Methods

### 2.1. Study Population

A case-control study was conducted, and participants were consecutively recruited from the Neurology Outpatient Clinic of the Universidad Autonoma of Nuevo Leon, Monterrey, Mexico, from July 2009 to July 2010. Inclusion criteria were subjects with a diagnosis of AD according to the DSM IV and NINCDS-ADRA Criteria [11]. Healthy controls (HC) matched for age (±3 years), sex, and vascular risk factors were also recruited. Subjects who did not attend the imaging study appointment and those with severe functional impairment (inability to attend the appointments), psychiatric disorder (at the time of AD diagnosis), history of myocardial infarction, angina pectoris, transient ischemic attack or stroke, severe carotid stenosis (>50%), and respiratory or cardiac conditions were excluded from the study. Informed consent was obtained for every participant in the study (and the family caregivers in AD participants) and this study was approved by the Local Ethics Committee.

### 2.2. Magnetic Resonance Imaging (MRI), Carotid Ultrasound Study, and Blood Samples

To evaluate for possible confounding factors, an MRI study was made to assess for white matter damage (leukoaraiosis) and a carotid ultrasound study to assess for carotid plaques and an intima-media thickness (IMT). In addition, blood samples were obtained for every participant and processed for common vascular risk biomarkers: cholesterol, low-density lipoprotein, high-density lipoprotein, triglycerides, C-reactive protein, and homocysteine.

### 2.3. Clinical Data, Cognitive and Depression Test

Demographic and vascular risk factors data were obtained through a structured clinical interview, and participants were classified as having diabetes mellitus (DM) if they were using antidiabetic drugs or with self-reported history; also hypertension and dyslipidemia status were assessed similarly. Cognitive function was evaluated with Mini-Mental State Examination test (MMSE) Spanish version [12, 13] and the Geriatric Depression Scale in its 15-item Spanish version was applied to screen for depression symptoms [14].

### 2.4. Cerebral Vasomotor Reactivity Protocol and TCD Study

CBFVs and Pulsatility Indices (PIs) were evaluated with a 2 MHz Probe TCD (Rimed; Smart-Lite SL-1 TCD System) with the participant in a supine position; measurements were taken in the left MCA by the same examiner at an insonation deep of 55 millimeters through the temporal window. After baseline CBFVs and PIs measurement, a CVMR test was made asking the patient to inhale a 7% CO2-air mixture for 5 minutes according to Deplanque et al. [15]. After the CO2 inhalation, CBFV and PIs measurement were repeated. Variables collected were CBFV, systolic CBFV (CBFV-S), diastolic (CBFV-D), and mean (CBFV-M). PIs were manually calculated using the formula of Gosling and King [16], CVMR was calculated as CBFV post CO2 - CBFV at baseline/CBFV at baseline x 100, and calculations were made for all velocities. Mean arterial pressure (MAP) was obtained prior to and after the CVMR test. All tests were conducted in the vascular research laboratory of the Neurology Department at a constant temperature and with a 10-minute resting period before initial CBFV evaluation.

### 2.5. Statistical Analyses

Comparisons of continuous variables were analyzed with a Mann–Whitney U test and a chi-square test was used for categorical variables. A multivariate analysis was made to adjust for age and sex. Finally, a Spearman correlation test between MMSE results, CBFVs difference, and %CVMRs was performed. Results were considered significant if p=<0.05. All statistical analysis was made using SPSS v22.0.

## 3. Results 

### 3.1. Participant General Characteristics

A total of 51 subjects, 26 participants with AD and 25 healthy, were initially included; however, 6 controls who did not attend the initial carotid ultrasound and MRI study appointment were excluded. General demographic, cognitive, and depression test results are shown in Table 1. There were no statistically significant differences in cardiovascular risk factors or GDS scores between both groups. There was a significant difference in MMSE scores between AD participants and HC.

### 3.2. MRI, Carotid Doppler Ultrasound Study, and Vascular Biomarkers Results

There were no differences in the severity of white matter damage between the groups. In addition, there were no significant differences in the presence of carotid plaques or intima-media thickness estimate. There were no statistically significant differences between AD participants and HC in cholesterol, LDL, HDL, triglycerides, C-reactive protein, and homocysteine levels. Full results of MRI, carotid doppler, and blood samples are shown in Table 1.

### 3.3. Baseline CBFV and PIs Results

Baseline CBFVs in MCA were obtained from all participants.  Table 2 shows results obtained for both groups. There were no significant differences in baseline BP (blood pressure), PIs, or any of the CBFVs between AD participants or HC.

### 3.4. Cerebral Vasomotor Reactivity Test Results

All recruited participants were able to perform the entire CVMR test. There were significant absolute differences in CBFVs between AD and HC groups after the CO2 test, and calculated %CVMR for changes in diastolic, systolic, and mean CBFVs were statistically significant between both groups; however after adjusting for age, sex, hypertension, and DM, only changes in diastolic CVMR remained statistically significant (7.45±18.25 versus 23.29±17.48, p<0.05). Full results are shown in Table 2.

### 3.5. MMSE Cognitive Test and CBFV

There was a positive correlation between MMSE results and changes in CBFV-S (*ρ*=0.339, p=0.023), CBFV-D (*ρ*=0.422, p=0.004), and CBFV-M (*ρ*=0.299, p=0.046). When correlating MMSE with %CVMRs, only %CVMR-D (*ρ*=0.337, p=0.023) remained statistically significant.

## 4. Discussion

### 4.1. Main Findings

This study shows that participants with AD have smaller changes in CBFVs than healthy controls matched for age, gender, and common vascular risk factors in response to an inhaled CO2 CVMR test, particularly in diastolic phase CBFV. Also, this decreased response may not be related to differences in common vascular risk biomarkers such as white matter damage, atherosclerotic disease, cholesterol, C-reactive protein, or homocysteine levels. As far as we know, these are the first results published from a Latin-American AD sample.

### 4.2. Baseline CBFVs

This study showed no baseline alteration in CBFVs or PIs. Similar results have been previously published; a study by Lee et al. [17] showed no differences in baseline CBF between AD participants and HC. Although a study by Vicenzini et al. [18] found that lower baseline CBFV and higher PI are present in AD participants, a recent meta-analysis of hemodynamic studies in demented subjects performed by Sabayan et al. showed a significant but small reduction in CBFVs and increased PIs in AD [19]. This cerebral hypoperfusion condition in patients with AD could be the result of small vessel damage driven by amyloid angiopathy, a landmark of AD brain findings among other alterations [20]. Increased PIs may be also explained by damage to cerebral microvasculature, leading to an increase in vascular resistance.

### 4.3. CVMR Alterations

CVMR attenuation in AD has also been shown in other studies; Abeelen et al. [21] showed a CVMR alteration in AD participants related to healthy controls in response to hypercapnia. Studies that used other imaging modalities such as BOLD-fMRI [22] also reported similar results. The fact that, in this study, only changes in diastolic phase CBFVs were statistically significant could be explained by the low sample size; however, the reason is unknown and future studies to address this observation are required. Impairment of CVMR has been proposed to be a consequence of multiple mechanisms, of which amyloid-*β* protein deposits in small cerebral vessels are the most important. This accumulation of amyloid-*β* leads to a decreased vasodilator synthesis driven by oxidative stress and disruption of blood-brain barrier with a subsequent perivascular edema that further reduces CVMR response, in addition to other glial and neuronal intrinsic pathological factors such as cholinergic dysfunction [4]. Pathophysiological mechanism pathways to vascular damage could be different in Latin-American population; a study made by O'Bryant et al. showed that Mexican American participants with AD could have a significantly different serum biomarker profile [23]; also the progression of these mechanisms is faster Latin-American subjects compared to non-Hispanic whites [24].

Vascular pathology plays a central role in the development and progression of Alzheimer's disease. These changes may appear before the clinical manifestation [25]; also people with Apolipoprotein E high risk alleles may have an impaired baseline vascular function [26]. Although the pathophysiological processes of these alterations in CVMR within the context of dementia and AD are not fully understood, it is well known that CVD and traditional vascular risk factors such as age, dyslipidemia, diabetes mellitus, hypertension, smoking, and metabolic syndrome can individually lead to alterations in CVMR, fostering the vascular dysfunction in AD [5]. A population-based study published by Wolters et al. revealed that subjects with higher CVMRs were less likely to develop AD (hazard ratio of 0.84) particularly those carrying APOe4 alleles (hazard ratio of 0.77) at 11.5 years of follow-up; these results give clinical usefulness to the measurement of CVMR in healthy individuals at risk [27].

### 4.4. MMSE and CVMR

This study showed a weak positive correlation between MMSE results and CBFVs change before and after CVMR test, although a study by Lee et al. [17] showed no correlation between MMSE and CVMR. Clinically, vascular dysfunction in AD results in further cognitive decline and functional impairment; a study made by Silvestrini et al. demonstrated a moderate correlation between both characteristics, showing that a breath holding index below 1 as a measure of CVMR is correlated with a progressive decline in MMSE scores [23]. Further studies are needed to measure the real magnitude of the correlation between MMSE and CVMR.

### 4.5. Limitations

This study had several limitations: first, we did not perform a measurement of end-tidal CO2 because of limited resources and we did not assess CVMR in hypocapnia condition. Also, AD diagnosis was clinical criteria-based, and severity of dementia was not directly assessed. Only MCA measurements were taken for feasibility purposes. DM as a cardiovascular risk factor was considered with only clinical history without considering blood sugar or HbA1c readings; thus there was not a distinction between those with controlled or uncontrolled DM and neither for hypertension or dyslipidemia. Weight/BMI and PAD were not assessed. Also, subjects with a GDS-15 score compatible with probable depression in the study appointment were not excluded. Moreover, 6 healthy control participants who did not attend the MRI appointment were excluded; however, baseline results showed no differences. Despite this, these results should be confirmed in a larger sample size.

## 5. Conclusions

Despite being an indirect measurement of overall vascular function, measurement of baseline CBFVs and CVMR responses in AD subjects with TCD is easy, safe, and cost-effective. Therefore, these virtues make this technique ideal for its use in the clinical setting of developing countries. This study adds strength to the general reproducibility of these results for the use of CVMR test in the clinical setting of AD with Latin-American samples.

## Figures and Tables

**Table 1:** Demographic, clinical, imaging, and blood sample characteristics of Alzheimer's disease and healthy control participants.

Characteristic	Alzheimer's disease (n=26)	Healthy controls (n=19)
**(a) Demographic and clinical**		

Age, median (range)*∗*	78(67-93)	78 (59-90)

Gender, fem (%)	21 (81%)	15 (79%)

Education years, median (range)	3(0-15)	6 (0-16)

Diabetes mellitus, n (%)	9(35%)	4(21%)

Hypertension, n (%)	13(50%)	8(42%)

Dyslipidemia, n (%)	5(19%)	9(47%)

Active smoking, n (%)	6(23%)	7(37%)

MMSE, media (±SD)	14.08(±5.80) ^†^	27±(3.20)

GDS, median (range)	4 (0-9)	3 (0-7)

**(b) MRI study and carotid ultrasound**		

Leukoaraiosis >5mm	6 (23%)	2 (11%)

Carotid plaques >30%	11 (42%)	6 (32%)

Intima-media thickness	0.902 (0.60 -2.0)	0.826 (0.6-1.10)

**(c) Blood samples**		

Total cholesterol	182.2 ± 36.4	197.6 ± 33.5

LDL	111.6 ± 31.5	129.8 ± 32.3

HDL	36.7 ± 11.5	38.2 ± 9.6

Triglycerides	164.7 ± 100.4	147.5 ± 56.9

CRP	3.7 ± 5.0	6.6 ± 9.1

Homocysteine	10.3 ± 2.9	8.9 ± 3.6

MMSE: Mini-Mental State Examination; BMI: body mass index; GDS: Geriatric Depression Scale 15 item version; MRI: magnetic resonance imaging; LDL: low-density lipoprotein; HDL: high-density lipoprotein; CRP: C-reactive protein.

**∗**Years.

^†^p < 0·05.

**Table 2:** Baseline, post-CO2 test cerebral blood flow velocities, and calculated CVMR.

	Healthy Controls (n=19)			Alzheimer's disease (n=26)		

Velocities	Baseline mean (±SD)	Post-CO2 mean (±SD)	Mean CVMR % (±SD)	Baseline mean (±SD)	Post-CO2 mean (±SD)	Mean CVMR % (±SD)

CBFV-S	69.90 (18.44)	82.64 (14.47)	21.17(17.03)	62.3619.83)	68.30(20.50)^†^	10.88(15.26)

CBFV-D	26.36 (7.26)	31.86 (7.42)	23.29(17.48)	25.348.21)	27.09(9.78)^†^	7.45(18.25)^††^

CBFV-M	49.00 (15.52)	57.70 (14.08)	20.88(14.97)	38.8814.95)	43.69(16.87)^†^	15.02(27.69)

CBFV-S: cerebral blood flow velocity-systolic; CBFV-D: cerebral blood flow velocity-diastolic; CBFV-M: cerebral blood flow velocity-mean; CVMR %: cerebral vasomotor reactivity as a percentage of change between baseline CBFV and post-CO2 test CBFV.

^†^p < 0·05: comparison of post-CO2 mean CBFV between AD and HC groups.

^††^p < 0.05: comparison of mean CVMR % between AD and HC groups.

## Data Availability

The data used for this study are not publicly available due to local ethic regulation and patient privacy consent.
